# Multiplexed Lateral Flow Test for Detection and Differentiation of *Cronobacter sakazakii* Serotypes O1 and O2

**DOI:** 10.3389/fmicb.2017.01826

**Published:** 2017-09-20

**Authors:** Eva J. Scharinger, Richard Dietrich, Tobias Wittwer, Erwin Märtlbauer, Kristina Schauer

**Affiliations:** ^1^Department of Veterinary Science, Faculty of Veterinary Medicine, Ludwig-Maximilians-Universität München Oberschleißheim, Germany; ^2^R-Biopharm AG Darmstadt, Germany

**Keywords:** *Cronobacter sakazakii*, multiplexed lateral flow immunoassay, PIF, food analysis and safety, serotyping

## Abstract

The ubiquitous and opportunistic pathogen *Cronobacter sakazakii* is responsible for severe meningitis, sepsis, and necrotizing enterocolitis in neonates and infants associated with ingestion of contaminated powdered infant formula (PIF). The current ISO method for isolation and detection of *Cronobacter* spp. is laborious, time-consuming and expensive. In this study, a multiplexed lateral flow test strip was developed to rapidly detect and simultaneously serotype O1 and O2 *C. sakazakii* serotypes. The assay is based on two monoclonal antibodies (MAb) that specifically bind to the lipopolysaccharides (LPS) of these pathogens. The test strip provides results very quickly; *C. sakazakii* could be detected in pure culture within 15 min with a sensitivity of 10^7^ CFU/ml. After non-selective enrichment for 18 h as low as one *Cronobacter* cell per g PIF could be detected. Moreover, the established lateral flow assay (LFA) offers excellent specificity showing no cross-reactivity with other *C. sakazakii* serotypes, *Cronobacter* species or *Enterobacteriaceae* tested. These characteristics, together with several advantages such as speed, simplicity in performance, low analysis cost, and no requirement of specialized skills or sophisticated equipment make the developed multiplexed LFA suitable for reliable detection and serotyping of *C. sakazakii* serotypes O1 and O2.

## Introduction

*Cronobacter* spp. are opportunistic foodborne pathogens and can cause severe infections in all age groups, with neonates being the most susceptible and affected group for invasive disease (Lai, 2001). However, the number of neonatal infections with *Cronobacter* caused by contaminated PIF might be underestimated globally (Tothova et al., 2011). The genus comprises of seven species among which *Cronobacter sakazakii* are primarily associated with neonatal infections such as necrotizing enterocolitis (NEC), bacteremia and meningitis resulting in a high mortality rate of 40–80% (Bowen and Braden, 2006; Jason, 2012). At present, the species *C. sakazakii* consists of five serotypes O1–O4 and O7 (Blažková et al., 2015). Predominantly the serotypes O1 and O2 are isolated from clinical as well as from food products, suggesting that some serotypes of *C. sakazakii* might be more virulent than others (Blažková et al., 2015; Yan et al., 2015a,b; Scharinger et al., 2016). Therefore, it is important for epidemiological studies to not only identify the genus and species but also the serotype of the pathogen.

Epidemiologically, neonatal *C. sakazakii* infections are linked to the consumption of contaminated powdered infant formula (PIF) (Iversen and Forsythe, 2004; Drudy et al., 2006; Craven et al., 2010). As *Cronobacter* spp. does not survive the standard pasteurization process, a *Cronobacter* contamination critical for causing disease can only occur during filling and packaging and/or during reconstitution of PIF (Nazarowec-White and Farber, 1997; Parra-Flores et al., 2015). Furthermore, a hallmark of *Cronobacter* spp. is its extreme desiccation resistance: this bacterium may be recovered even after 2.5 years from a dairy food, in contrast to other PIF-associated bacteria such *Salmonella enterica* serovar Enteritidis and *Escherichia coli* which were already undetectable after 15 months (Barron and Forsythe, 2007). Therefore, a microbial risk assessment for *E. sakazakii* and other bacteria in PIF was developed by the WHO/FAO.

The contamination of PIF with *Cronobacter* spp. is being monitored using optimized conventional microbiological methods published by the International Standards Organization (ISO) and the International Dairy Federation (IDF) (ISO, 2006). However, these methods are laborious and definitive identification of *Cronobacter* species–without serotyping–takes up to 6 days, because diverse enrichments and biochemical confirmation steps are required. Besides culture-based methods, other rapid, highly sensitive and specific approaches for *Cronobacter* detection such as fluorescence *in situ* hybridization (FISH) (Almeida et al., 2009), DNA microarray (Wang et al., 2009), polymerase chain reaction (PCR) (Zimmermann et al., 2014; Yan and Fanning, 2015; Zhou et al., 2016), and antibody-based immunoassays (Hochel and Škvor, 2009; Blažková et al., 2011; Park et al., 2012; Xu et al., 2014; Scharinger et al., 2016) have been reported. All these techniques require specialized equipment and trained staff, consequently, they become expensive and laboratory-based.

Ideally, a new and improved diagnostic tool to detect *Cronobacter* should be more rapid, reliable, efficient, inexpensive and user-friendly. Among the diagnostic tests, the immunochromatographic strip assays satisfy all these prerequisites and some lateral flow tests for different pathogenic bacteria or their agents have already been successfully developed in order to enhance food safety (Kawatsu et al., 2008; Fang et al., 2014; Cox et al., 2015; Wu et al., 2015; Raeisossadati et al., 2016). Therefore, the aim of this study was to develop a multiplexed lateral flow assay (LFA) for rapid detection and simultanous serotyping of *C. sakazakii* serotypes O1 and O2, based on the recently described highly serotype specific monoclonal antibodies (MAbs) (Scharinger et al., 2016). The ability of the established lateral flow test strip to specifically detect *C. sakazakii* was confirmed by analyzing artificially contaminated PIF samples after enrichment in buffered peptone water (BPW) in comparison to the standard ISO reference method. This new multiplexed LFA shows great promise to complement or replace the time-consuming and culture-based methods for *Cronobacter* detection and has a high potential for on-site detection.

## Materials and methods

### Bacterial strains

The bacterial strains used in this study are listed in Table 1. All strains were grown in Luria-Bertani (LB) medium at 37°C with constant shaking. For solid media, 15 g/l agar was added. To measure the number of colony forming units (CFU), bacteria were quantified by plating 10-fold serial dilutions on Chromogenic *Cronobacter* Isolation Agar (CCI, Oxoid) or LB agar plates. The genus, species and serotype of *Cronobacter* strains were identified according to the proposed PCR-based schema for *Cronobacter* spp. (Lehner et al., 2006; Stoop et al., 2009; Jarvis et al., 2011; Sun et al., 2012; Carter et al., 2013; Yan et al., 2015a).

**Table 1:** Reactivity of bacterial strains used in this study.

**Strain**	**Species**	**Origin**	**Source/reference**	**Serotype**	**Sandwich EIA^a^**	**Lateral flow assay^b^**
MHI 975	*C. sakazakii* ATCC 29544	Human	Iversen et al., 2007b, 2008	O1	+	+
MHI 996	*C. sakazakii*	Baby food	LMU	O1	+	+
MHI 21011	*C. sakazakii*	Baby food	LMU	O1	+	+
MHI 21038	*C. sakazakii* ATCC BAA 893-1	Milk powder	Mullane et al., 2008	O1	+	+
MHI 21086	*C. sakazakii*	Milk powder	LMU	O1	+	+
MHI 977	*C. sakazakii* NCTC 8155	Tin of dried milk	Iversen et al., 2008	O2	+	+
MHI 995	*C. sakazakii*	Baby food	LMU	O2	+	+
MHI 21003	*C. sakazakii*	Baby food	JLU	O2	+	+
MHI 21004	*C. sakazakii*	Baby food	JLU	O2	+	+
MHI 21122	*C. sakazakii* ATCC 12868	Unknown	Iversen et al., 2007a	O2	+	+
MHI 21006	*C. sakazakii*	Baby food	JLU	O3	−	−
MHI 21014	*C. sakazakii*	Baby food	LMU	O3	−	−
MHI 21051	*C. sakazakii*	Milk powder	LMU	O4	−	−
MHI 21170	*C. sakazakii*	Baby food	JLU	O4	−	−
MHI 21066	*C. sakazakii*	Milk powder	LMU	O7	−	−
MHI 21171	*C. sakazakii*	Baby food	JLU	O7	−	−
MHI 21097	*C. condimenti* LMG 26250^T^	Food	Joseph et al., 2012		−	−
MHI 21093	*C. dublinensis* subsp. *dublinensis* LMG 23823^T^	Milk powder	Iversen et al., 2007a		−	−
MHI 21094	*C. dublinensis* subsp. *lausannensis* LMG 23824^T^	Water	Iversen et al., 2007a	O2	−	−
MHI 21095	*C. dublinensis* subsp. *lactaridi* LMG 23825^T^	Milk powder	Iversen et al., 2007a	O1	−	−
MHI 21091	*C. malonaticus* DSM 18702	Human	Iversen et al., 2007a	O2	−	−
MHI 21096	*C. muytjensii* DSM 21870	Unknown	Iversen et al., 2007a	O2	−	−
MHI 21213	*C. muytjensii* E888	Milk powder	UZ	O1	−	−
MHI 21026	*C. turicensis* 3032 LMG 23827^T^	Neonate	Iversen et al., 2007a; Stephan et al., 2011	O1	−	−
MHI 21049	*C. turicensis* E625	Baby food	UZ	O3	−	−
MHI 21092	*C. universalis*				−	−
MHI 21103	*Franconibacter helveticus* LMG 23732^T^	Fruit powder	Stephan et al., 2014		−	−
MHI 21105	*Franconibacter pulveris* LMG 24057^T^	Fruit powder	Stephan et al., 2014		−	−
MHI 21104	*Siccibacter turicensis* LMG 23730^T^	Fruit powder	Stephan et al., 2014		−	−

a *Using specific MAbs 1C4 (O1), 2F8 (O2) (Scharinger et al., 2016)*.

b *Reactivity of overnight culture (LB medium)*.

*(+) Positive result*.

*(−) Negative result*.

*LMU Chair of Hygiene and Technology of Milk, Ludwig-Maximilians-Universität München, Munich, Germany*.

*JLU Institute of Veterinary Food Science, Justus-Liebig-Universität Giessen, Germany*.

*UZ Institute of Food Safety and Hygiene, University of Zürich, Zurich, Switzerland*.

### Monoclonal antibodies

MAbs 1C4 and 2F8 specific to *C. sakazakii* serotypes O1 and O2 respectively were described in a previous study (Scharinger et al., 2016).

### Preparation of the multiplexed lateral flow test strip

**Figure 1:**
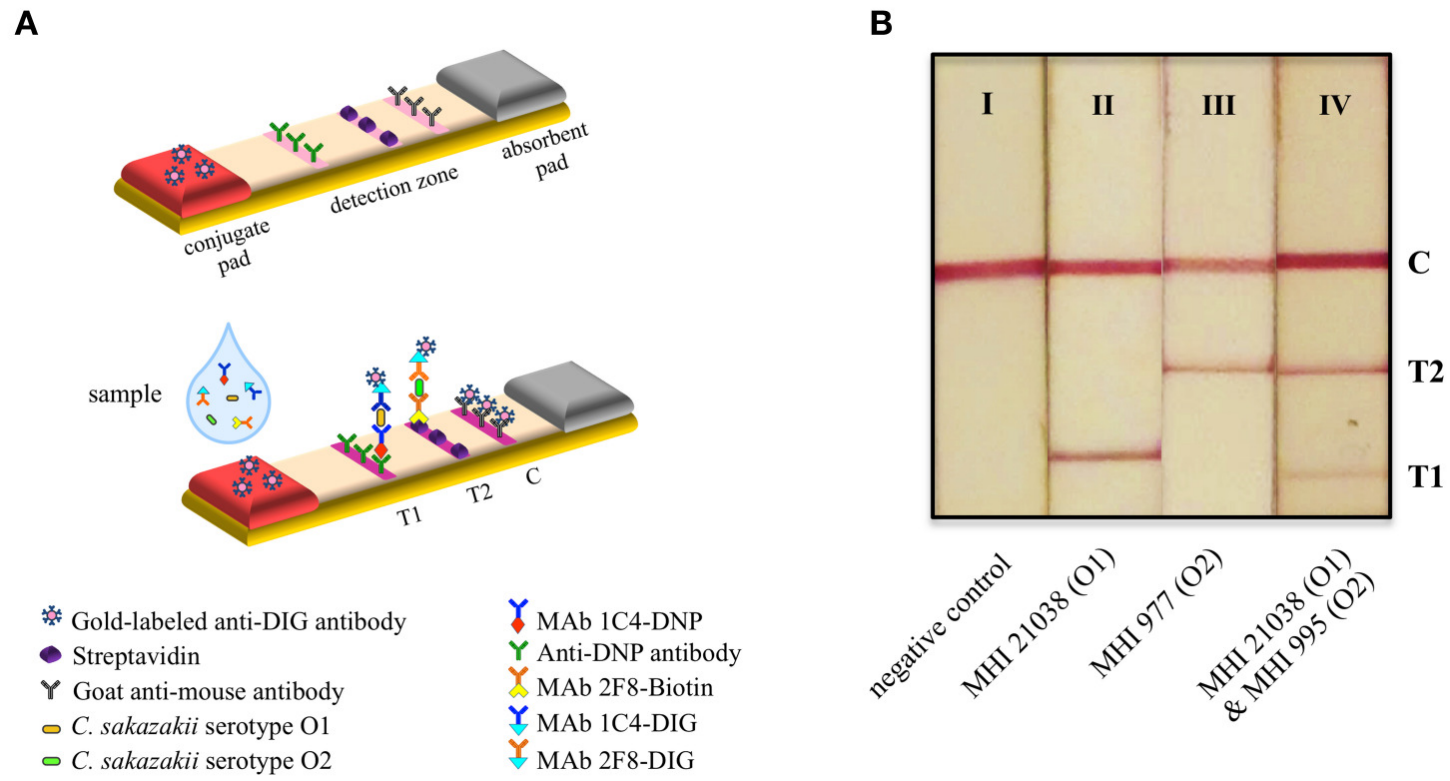
Principle and application of the multiplexed lateral flow test strip. **(A)** Schematic representation of the developed immunochromatographic assay. **(B)** Lateral flow test strips used for detection of *C. sakazakii* in inoculated PIF samples. Interpretation of the LFA results: I, negative (only one red line for the control line), II, and III, positive for *C. sakazakii* serotypes O1 and O2 (two red lines at the readout are: control line and test line 1 or test line 2, respectively), IV, positive for both *C. sakazakii* serotypes O1 and O2 (three red lines at the readout are: control line and two test line 1 and 2). C, control line; T1 and T2, test lines 1 and 2.

The LFA was carried out in a dipstick format (Figure 1A) which is typically composed of four parts: a sample pad for loading of the sample, a conjugate pad with the deposited detection molecule, a reaction nitrocellulose membrane for formation of the detectable colored lines and finally, an absorbent pad enhancing the capillary driving force and adsorbing non-reacting substances. In our developed lateral flow test strip, the sample pad was omitted and replaced by a longer conjugate pad. All parts are connected to each other and incorporated in the plastic backing card to ensure robustness of the LFA. The strips were prepared by the R-Biopharm AG in Germany. The detection zone of the strip is composed of three test lines, dispensed by an automatic dispenser on the nitrocellulose membrane (FF120 HP, Whatman), and contained (1) immobilized anti-dinitrophenyl (DNP) antibodies and (2) streptavidin as test lines for *C. sakazakii* serotype O1 and O2, respectively, and (3) goat-anti-mouse antibodies (Sigma-Aldrich, Steinheim, Germany) as a control line. As tracer conjugate, an anti-digoxigenin (DIG) antibody (Sigma-Aldrich, Steinheim, Germany) was conjugated to colloidal gold particles (diameter = 40 nm) and sprayed onto the glass fiber conjugate pad (SureWick, Merck Millipore). To achieve a sandwich format for the LFA, *C. sakazakii* serotype O1 and O2 specific antibodies were either conjugated to DNP and DIG (MAb 1C4 specific for *C. sakazakii* serotype O1) or to biotin and DIG (MAb 2F8 specific for *C. sakazakii* serotype O2). The mixed DNP- and biotin-labeled antibodies MAb 1C4-DNP (3.90 μg/ml) and MAb 2F8-biotin (2.49 μg/ml) were combined in dilution buffer (100 mM NaCl, 0.5% BSA, 0.5% Tween 20, 0.049% sodium azide in 50 mM phosphate buffer; pH 8.0) and designated as solution A, while DIG-labeled antibodies (MAb 1C4 5.31 μg/ml and MAb 2F8 2.85 μg/ml) represent solution B.

During the experiment, 100 μl of the bacterial suspension or pre-enriched baby food-sample was mixed with 400 μl dilution buffer. Fifty microliters of this mixture was combined with 50 μl of solution A and 50 μl of solution B. After 5 min of incubation at room temperature, 100 μl of this mixture was applied to the strip. The test results could be observed within 15 min of applying the sample. The signal intensities of the test and control lines were evaluated visually.

### Inclusivity and exclusivity tests

The reactivity and specificity of the used MAbs 1C4 and 2F8 has been determined in a previous study by testing more than 100 *Cronobacter* spp. and other *Enterobacteriaceae* using EIAs (Scharinger et al., 2016). For this reason, only a few representative strains were used in this study to verify the specificity of the established LFA (Table 1). In order to investigate the inclusivity of the developed immunochromatographic strip, the reactivity of five *C. sakazakii* serotype O1 and five *C. sakazakii* serotype O2 strains was analyzed with the lateral flow test strip. The exclusivity of the LFA was tested by analyzing two strains each of the *C. sakazakii* serotypes O3-O4 and O7 as well as other *Cronobacter* species and foodborne pathogens often associated with *C. sakazakii* in PIF (Table 1). The tested strains were cultured in LB medium overnight to an average bacterial cell count of 10^9^ CFU/ml. Then, the overnight cultures were mixed with dilution buffer, solutions A and B as described above, before applying 100 μl of this mixture directly to the lateral flow test strips.

### Detection limit

The limit of detection (LOD) of the LFA was determined by analyzing overnight culture of five strains of *C. sakazakii* serotype O1 and O2 each and with *C. sakazakii* inoculated follow-on formula milk powder. The pure bacterial cultures were tested in the LFA with and without 10-fold serial dilutions and the *Cronobacter* cell counts were determined in parallel as described above. The used *C. sakazakii* strains are listed in Table 1. To determine the LOD of the LFA in PIF, 10 g of follow-on formula milk powder inoculated with *C. sakazakii* strains MHI 21011 (serotype O1) and MHI 977 (serotype O2) at a level of 1 CFU per g were analyzed by the LFA. These samples were enriched in 90 ml BPW in accordance with the ISO standard method by incubating at 37°C for 20 h without agitation. PIF in BPW without bacteria served as a negative control. After incubation times of 8, 12, 16, 18, and 20 h, sample aliquots (three replicates for each sample and time point) were analyzed by the lateral flow test strip. In parallel, the exact CFU of each sample was determined according to the ISO method on CCI agar.

### Evaluation of the test with standard reference material

nutriCert®-*Cronobacter* spp. in milk powder (25 g) reference material was obtained from the DRRR (Deutsches Referenzbüro für Ringversuche und Referenzmaterialien; German reference office for food proficiency testing and reference materials, Kempten, Germany). The standard reference material included two positive samples, each contaminated with *C. sakazakii* ATCC 29544 (serotype O1), and one negative sample without *Cronobacter*. Each sample was dissolved 1/10 in 225 ml BPW, incubated at 37°C without agitation for 18 h and then analyzed by the LFA. For control purposes, the *Cronobacter* cell counts were determined in parallel as described above.

### Artificially spiked baby food experiments

Three different kinds of dried baby food, including two initial formulas (from birth on), two follow-on formulas (from 6th month on) and two plant-based baby food (mash) were obtained from a local German drugstore and tested negative for the presence of *Cronobacter* spp. using the detection method ISO/TS 22964:2006. For spiking experiments, each sample (10 g) was mixed with 90 ml BPW and artificially contaminated with three different strains of *C. sakazakii* serotype O1 (MHI 996, MHI 21011, MHI 21038) and O2 (MHI 977, MHI 995, MHI 21122). The inoculation of samples was carried out with 1 ml of a dilution of the *C. sakazakii* overnight culture corresponding to 1 or 10 CFU. In parallel, the real CFU/ml of each inoculum was determined by plating on CCI agar. Artificially contaminated samples were enriched in accordance with the ISO standard. After 18 h of non-selective enrichment as well as after a subsequent selective enrichment in mLST/vancomycin broth (20 h), samples were analyzed by the lateral flow test strip. In parallel, the CFU of each sample was determined by plating 10-fold serial dilutions on CCI agar.

### Detection of *C. sakazakii* in naturally contaminated samples

In total five *Cronobacter*-positive, in Mossel-Bouillon enriched samples were provided from a local PIF producing factory. Three environmental swabs, consisted of two floor samples before cleaning as well as one after cleaning, and two samples from the produced PIF were analyzed with the Assurance GDS™ *Enterobacter sakazakii* (BioControl, Bellvue, USA) by the factory laboratory. These enriched samples were directly applied to the lateral flow test strips according to the established protocol.

## Results

### Detection principle of the developed multiplexed lateral flow test strip

The developed LFA is based on a sandwich-EIA format as illustrated in Figure 1A. In contrast to the usual LFA design in which gold-labeled antigen-specific antibodies serve as detection reagent, here a universally applicable experimental set-up was realized. This approach is based on the use of hapten-specific antibodies or reagents (streptavidin) for both capturing the immune complexes on the test membrane and visualizing bound immune complexes. This toolbox approach offers high-flexibility and broad versatility to the simple and fast realization of LFAs as the often poorly reproducible gold-labeling of the primary, antigen-specific antibodies is avoided. Prior to being applied to the lateral flow test strip, the sample was pre-incubated with solution A and B containing differently labeled anti-*C. sakazakii* antibodies (see Material and Methods). During the incubation of 5 min, a sandwich complex consisting of the bacterial cell and the different labeled antibodies forms. In the case of *C. sakazakii* serotype O1, DIG-, and DNP-labeled antibodies (MAbs 1C4) bind to the LPS structures on the bacterial surface, whereas in the case of *C. sakazakii* serotype O2 the sandwich complex is composed of the bacteria bound to a DIG- and a biotin-labeled antibodies (MAbs 2F8). After being applied to the lateral flow strip, the complex is bound by either the anti-DNP antibodies (test line 1) or streptavidin (test line 2) (Figure 1A). Gold labeled anti-DIG antibodies from the conjugation pad will bind to the DIG-labeled anti-*Cronobacter* antibody and lead to the formation of a visible red band. Unbound anti-DIG antibodies will be immobilized on the control line (goat-anti-mouse antibody). Coloration of the control line indicates the correct performance of the LFA. The excess fluid and any unreacted substances are absorbed by the absorbent pad. Observation of both a colored test line 1 or test line 2 and a colored control line on the membrane is a positive result indicating the presence of *C. sakazakii* serotype O1 or *C. sakazakii* serotype O2 in the sample. If both *Cronobacter* serotypes are present in a sample three colored bands would be obtained. A single colored control line represents a negative result (Figure 1B). In addition, the stability and non-bleaching of the colloidal gold-tracer enables the conservation of results for ≥10 years, provided that the fluid flow on the strip was interrupted after 15 min of reaction time.

### Specificity and sensitivity of the lateral flow test strip

To determine the specificity of the LFA, inclusivity and exclusivity tests were performed with pure bacterial cultures. For this purpose, five strains of *C. sakazakii* serotype O1 and O2 each were analyzed as well as two strains of each *C. sakazakii* serotypes O3, O4, O7, and ten *Cronobacter* spp.-strains, including *C. condimenti, C. dublinensis, C. malonaticus, C. muytjensii, C. turicensis*, and *C. universalis*. Three additional strains of the *Enterobacteriaceae* family, namely *Franconibacter helveticus, Franconibacter pulveris*, and *Siccibacter turicensis* were also tested because they often simultaneously occur in the same PIF. The CFU of the applied bacterial cultures ranged between 10^8^ and 10^9^ CFU/ml. For *C. sakazakii* serotype O1- and O2-strains (*n* = 10) the corresponding serotype specific colored test line and the colored control line appeared in all cases within 15 min after application of the sample to the strip. Thus, the serotypes of the *C. sakazakii* strains were identified correctly. For all other tested strains (*n* = 19) only the colored control line appeared on the test strip. Thus, no indication for false positive or false negative results was obtained (Table 1).

**Figure 2:**
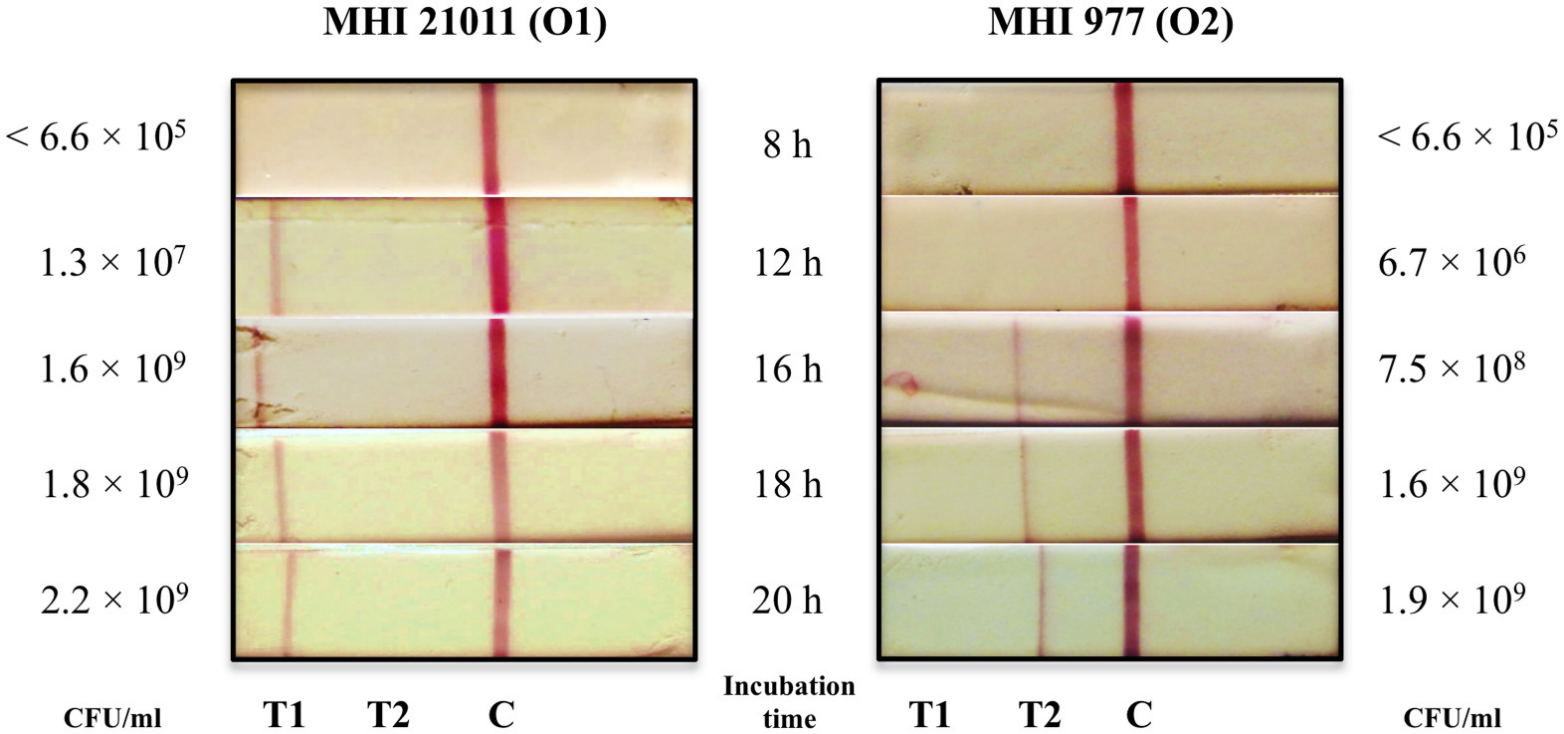
Detectability of *C. sakazakii* serotypes O1 and O2 after non-selective enrichment. PIF samples (10 g) were inoculated with 10 CFU of either strain MHI 21011 (*C. sakazakii* serotype O1) or MHI 977 (*C. sakazakii* serotype O2) and enriched in BPW. The samples were collected at 8, 12, 16, 18, and 20 h and analyzed by the LFA. The bacterial cell counts (CFU/ml) of the samples were determined by plating onto CCI agar.

The sensitivity of the multiplexed lateral flow test strip was evaluated using the spiked PIF format. For this purpose, PIF samples were enriched for 8, 12, 16, 18, and 20 h in BPW and then analyzed by the LFA. In parallel, the number of CFU was determined by plating serial dilutions on CCI agar. As shown in Figure 2, after 8 h of enrichment, the bacterial concentration of *C. sakazakii* strains MHI 21011 (O1) and MHI 977 (O2) did not exceed 7 × 10^5^ CFU/ml and were not detectable with the lateral flow test strip. After 12 h, strain MHI 21011 (O1) reached a bacterial cell count of 1.3 × 10^7^ CFU/ml and was detectable by clearly visible test line 1 on the lateral flow test strip, while at the same time point strain MHI 977 (O2) was not detectable due to the lower cell-count of 6.7 × 10^6^ CFU/ml. After 16 h of enrichment, strain MHI 977 (O2) with a bacterial cell count of 7.5 × 10^8^ CFU/ml was also detectable. Longer enrichment of up to 20 h did not improve the reactivity of the test (Figure 2).

### Detection of *C. sakazakii* in reference material

nutriCert®-*Cronobacter* spp. in milk powder reference material (three samples at 25 g) was subjected to the cultured-based ISO detection procedure. After a non-selective enrichment step, samples were directly analyzed by the LFA and plated onto CCI agar to determine the CFU/ml. *C. sakazakii* serotype O1 could be detected in PIF sample 1 and PIF sample 3 at a bacterial cell count of 1.2 × 10^9^ CFU/ml. PIF sample 2 (negative control) was negative in the lateral flow test strip which was confirmed by the absence of bacterial growth on CCI agar (Table 2).

**Table 2:** Analysis of reference material “nutriCert®-*Cronobacter* spp. in milk powder” after non-selective and selective enrichment.

**PIF**	**Lateral flow assay**		**CFU/ml**^**c**^	
	**BPW^a^**	**mLST/vancomycin^b^**	**BPW^a^**	**mLST/vancomycin^b^**
Sample 1	+	+	1.2 × 10^9^	2.0 × 10^8^
Sample 2	−	−	−	−
Sample 3	+	+	1.2 × 10^9^	2.2 × 10^8^

*Both LFA and the ISO standard method (ISO/TS 22964:2006) were applied*.

*(+) Positive result*.

*(−) Negative result*.

a *Enriched BPW as sample (non-selective enrichment)*.

b *Enriched mLST/vancomycin broth as sample*.

c *Growth on CCI agar after non-selective and selective enrichment*.

### Detection of *C. sakazakii* in artificially contaminated food samples

**Figure 3:**
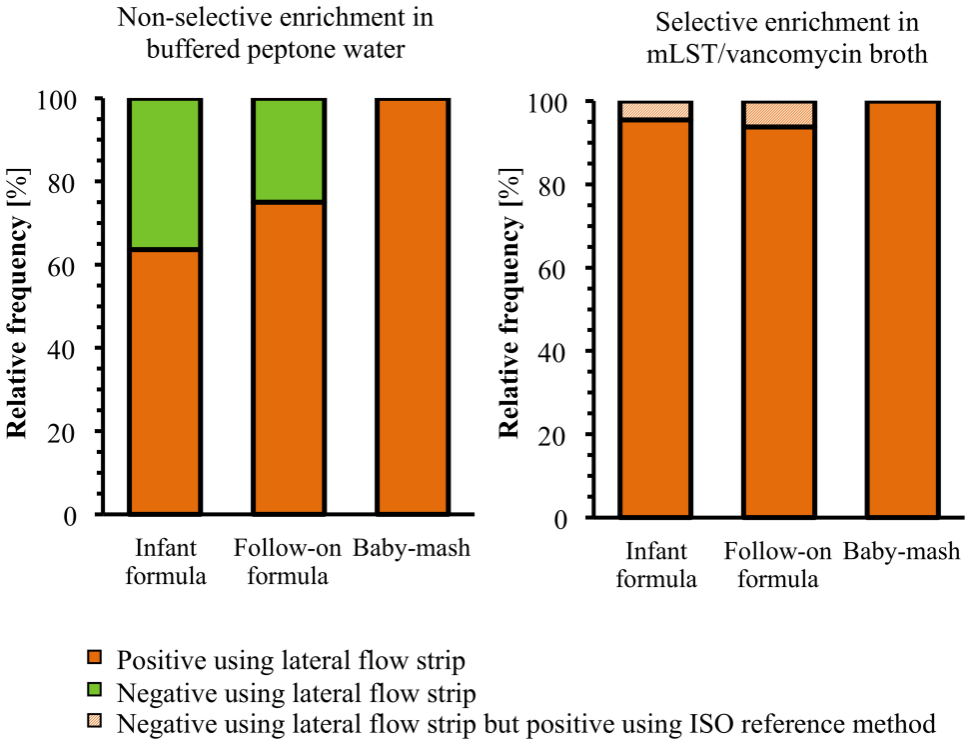
Overview of the LFA results obtained for artificially contaminated baby food samples (*n* = 58) after non-selective and selective enrichment in comparison to the ISO standard detection method.

Different commercially available infant food formulas, including initial milk formula, follow-on formula and plant-based baby food (mash) from two different manufacturers were chosen to test the applicability of the developed multiplexed LFA for the reproducible and sensitive detection of *C. sakazakii*. Samples of 10 g were dissolved in 90 ml BPW and each was subsequently inoculated with a strain of three different *C. sakazakii* serotype O1- (MHI 996, MHI 21011, MHI 21038) and three *C. sakazakii* serotype O2- (MHI 977, MHI 995, MHI 21122) strains. Except for strain MHI 21122 (origin unknown), all strains were originally isolated from baby food or milk powder/dried milk and thus were used for the artificial contamination of the infant food formulas in order to mimic natural conditions. Considering the zero tolerance limit laid down within the EU for *Cronobacter* in PIF (Anomymous, 2007), extreme low inoculum concentrations of one and ten bacterial CFU per 10 g milk powder were chosen to spike the samples prior to enrichment according to the ISO standard method. After non-selective pre-enrichment in BPW (18 h) and a subsequent selective enrichment in mLST/vancomycin broth (20 h), samples were applied to the lateral flow test strip and in parallel plated onto CCI agar to determine the bacterial cell count. Infant food formula samples without *Cronobacter* contamination served as negative controls and did not produce any false positive results in the LFA analyses. During validation study it turned out that 14 intrinsically with *Cronobacter* spiked PIF samples were tested negative both in LFA and by the ISO standard method and thus were excluded from the further statistical analysis (Figure 3). All these PIF samples has been spiked with low bacterial concentrations ranging from 0.8 to 3.1 CFU/10 g PIF.

In the contaminated initial milk formula, both *C. sakazakii* serotypes O1 and O2 could be detected in 63.6% (*n* = 7) of the spiked samples after the non-selective enrichment using the LFA (Figure 3). After a subsequent selective enrichment in mLST/vancomycin broth, 90.9% (*n* = 10) of all samples inoculated with *C. sakazakii* serotype O1 were tested positive. One false negative result was obtained for samples inoculated with 1 CFU of *C. sakazakii* MHI 21038 strain (Table 3). For *C. sakazakii* serotype O2, 100% (*n* = 11) of the samples tested positive after selective enrichment in mLST/vancomycin broth. Based on the CFU number of *Cronobacter* determined from the enriched cultures, most of the LFA-negative samples had not reached a bacterial concentration of >6.7 × 10^6^ CFU/ml. Thus, the lowest bacterial concentration that the LFA was able to detect was as low as 3 × 10^7^ CFU/ml (O1 serotype) and 1.3 × 10^7^ CFU/ml (O2 serotype) (Table 3).

**Table 3:** Lateral flow test results of artificially contaminated dried baby food (*n* = 72) after non-selective and selective enrichment in comparison to the ISO detection method (ISO/TS 22964:2006).

**Strain**	**Sample**	**CFU/10 g PIF^a^**	**CFU/ml after 18 h^b^**	**Lateral flow assay**		**Growth on CCI agar^e^**
				**BPW^c^**	**mLST/vancomycin^d^**	
**INITIAL MILK FORMULA (FROM BIRTH ON)**						
***C. sakazakii*** **serotype O1**						
MHI 996	Supplier A	2	6.7 × 10^6^	−	+	+
	Supplier A	20	3.6 × 10^7^	+	+	+
	Supplier B	2	<6.7 × 10^6^	−	+	+
	Supplier B	20	3.0 × 10^7^	+	+	+
MHI 21011	Supplier A	2.4	3.0 × 10^8^	+	+	+
	Supplier A	24	4.6 × 10^8^	+	+	+
	Supplier B	2.4	9.2 × 10^7^	+	+	+
	Supplier B	24	2.9 × 10^8^	+	+	+
MHI 21038	Supplier A	1.3	<6.7 × 10^6^	−	−	−
	Supplier A	13	1.3 × 10^9^	+	+	+
	Supplier B	0.7	<6.7 × 10^6^	−	−	+
	Supplier B	7	<6.7 × 10^6^	−	+	+
***C. sakazakii*** **serotype O2**						
MHI 977	Supplier A	2.5	1.3 × 10^7^	+	+	+
	Supplier A	25	7.3 × 10^7^	+	+	+
	Supplier B	2.5	1.3 × 10^7^	−	+	+
	Supplier B	25	7.9 × 10^7^	+	+	+
MHI 995	Supplier A	0.8	<6.7 × 10^6^	−	−	−
	Supplier A	8	<6.7 × 10^6^	+	+	+
	Supplier B	0.8	1.3 × 10^7^	+	+	+
	Supplier B	8	5.7 × 10^8^	+	+	+
MHI 21122	Supplier A	1	<6.7 × 10^6^	−	+	+
	Supplier A	10	6.7 × 10^6^	−	+	+
	Supplier B	1	1.3 × 10^7^	−	+	+
	Supplier B	10	7.7 × 10^7^	+	+	+
**FOLLOW-ON FORMULA (FROM 6TH MONTH ON)**						
***C. sakazakii*** **serotype O1**						
MHI 996	Supplier A	0.8	5.3 × 10^7^	−	+	+
	Supplier A	7.9	1.3 × 10^7^	+	+	+
	Supplier B	1.1	7.9 × 10^7^	+	+	+
	Supplier B	11	3.1 × 10^8^	+	+	+
MHI 21011	Supplier A	1.3	<6.7 × 10^6^	−	−	−
	Supplier A	13	<6.7 × 10^6^	−	+	+
	Supplier B	0.8	<6.7 × 10^6^	−	−	−
	Supplier B	8	2.0 × 10^8^	+	+	+
MHI 21038	Supplier A	1	6.7 × 10^6^	+	+	+
	Supplier A	10	1.6 × 10^7^	+	+	+
	Supplier B	1.8	<6.7 × 10^6^	−	+	+
	Supplier B	13	1.3 × 10^9^	+	+	+
***C. sakazakii*** **serotype O2**						
MHI 977	Supplier A	1	<6.7 × 10^6^	−	−	−
	Supplier A	10	1.9 × 10^8^	+	+	+
	Supplier B	1.1	3.0 × 10^8^	+	+	+
	Supplier B	11	3.0 × 10^8^	+	+	+
MHI 995	Supplier A	0.9	<6.7 × 10^6^	−	−	−
	Supplier A	8.7	6.7 × 10^6^	+	+	+
	Supplier B	0.6	<6.7 × 10^6^	−	−	−
	Supplier B	5.6	6.2 × 10^8^	+	+	+
MHI 21122	Supplier A	0.3	<6.7 × 10^6^	−	−	−
	Supplier A	3.1	<6.7 × 10^6^	−	−	−
	Supplier B	0.4	<6.7 × 10^6^	−	−	−
	Supplier B	4	<6.7 × 10^6^	−	−	+
**PLANT-BASED BABY FOOD (MASH)**						
***C. sakazakii*** **serotype O1**						
MHI 996	Supplier A	1.4	<6.7 × 10^6^	−	−	−
	Supplier A	14	1.3 × 10^9^	+	+	+
	Supplier B	1.1	<6.7 × 10^6^	−	−	−
	Supplier B	11	1.8 × 10^9^	+	+	+
MHI 21011	Supplier A	1.3	1.2 × 10^8^	+	+	+
	Supplier A	13	2.1 × 10^8^	+	+	+
	Supplier B	0.8	<6.7 × 10^6^	−	−	−
	Supplier B	8	7.2 × 10^8^	+	+	+
MHI 21038	Supplier A	1	1.0 × 10^9^	+	+	+
	Supplier A	10	9.3 × 10^8^	+	+	+
	Supplier B	1.8	6.7 × 10^8^	+	+	+
	Supplier B	18	8.0 × 10^8^	+	+	+
***C. sakazakii*** **serotype O2**						
MHI 977	Supplier A	0.7	5.2 × 10^8^	+	+	+
	Supplier A	6.7	6.8 × 10^8^	+	+	+
	Supplier B	1.3	<6.7 × 10^6^	−	−	−
	Supplier B	13	1.8 × 10^8^	+	+	+
MHI 995	Supplier A	1	4.8 × 10^8^	+	+	+
	Supplier A	9.5	7.6 × 10^8^	+	+	+
	Supplier B	0.9	1.6 × 10^8^	+	+	+
	Supplier B	9.2	4.6 × 10^8^	+	+	+
MHI 21122	Supplier A	1.2	8.1 × 10^8^	+	+	+
	Supplier A	12	9.3 × 10^8^	+	+	+
	Supplier B	1.4	2.4 × 10^8^	+	+	+
	Supplier B	14	6.1 × 10^8^	+	+	+

*(+) Positive result*.

*(−) Negative result*.

a *Real CFU/ml in inoculum*.

b *CFU/ml after non-selective enrichment in BPW*.

c *Enriched BPW as sample*.

d *Enriched mLST/vancomycin broth as sample*.

e *Growth on CCI agar after selective enrichment in mLST/vancomycin broth (ISO/TS 22964:2006) (ISO, 2006)*.

Applying samples of follow-on formula spiked with different *C. sakazakii* serotype O1-strains to the test strip, the following results were obtained: 70% (*n* = 7) tested positive after non-selective enrichment and 100% (*n* = 10) after selective enrichment in mLST/vancomycin broth. In contrast, *C. sakazakii* serotype O2 was detected in 83.3% (*n* = 5) of artificially contaminated samples after non-selective enrichment and no improvement was observed after the additional selective enrichment. Bacterial cell counts as low as 6.7 × 10^6^ CFU/ml were detectable by the LFA in follow-on formula (Table 3). One sample, inoculated with low bacterial cell counts (Table 3, Figure 3) reacted positive using the ISO standard method but negative in the LFA. In this case, the CFU number of ≥10^8^, characteristically obtained on average in mLST/vancomycin broth (Table 2), was not reached even after the selective enrichment.

For baby food samples such as plant-based mashes, that were spiked with the *C. sakazakii* serotypes O1 and O2, the LFA detected *Cronobacter* in 100% of the samples and in all cases already after non-selective enrichment (Figure 3). The lowest cell counts for *Cronobacter* in enriched baby food that could be detected by the developed LFA ranged from 1.2 to 1.6 × 10^8^ CFU/ml (Table 3). In contrast to the above described milk formula, a consistent growth of *Cronobacter* was observed in these plant-based baby foods (mash). In all spiked mash samples growth of *Cronobacter* could be confirmed, the CFU ranged from 1.2 × 10^8^ to 1.8 × 10^9^ CFU/ml after 18 h of non-selective enrichment (Table 3). Thus, identical results were obtained for the LFA and the ISO method (Figure 3).

### Detection of *C. sakazakii* in naturally contaminated factory samples

Naturally contaminated environment and food samples (*n* = 5) were obtained from a PIF-producing factory and analyzed to test the applicability of the multiplexed LFA in routine testing. The commercial diagnostic system Assurance GDS™ *E. sakazakii*, which is routinely used in the PIF-producing factory, gave *Cronobacter* spp. positive results for two of three environmental swabs (floor samples before cleaning) and both PIF samples. The third environmental swab, taken from the floor after the cleaning, did not test positive. Identical results were obtained with the developed multiplexed LFA using the same samples enriched in Mossel-Bouillon. In all positive samples *C. sakazakii* serotype O1 could be identified by the developed LFA.

## Discussion

With respect to the severe infections caused by *Cronobacter* spp. and manifested as necrotizing enterocolitis, sepsis or meningitis, a zero tolerance for the presence of *Cronobacter* spp. in PIF has been laid down within EU (FAO/WHO, 2007; VO(EU)365/2010, 2010). However, due to the ubiquitous character of this pathogen, PIF contamination during manufacture process, in households and hospitals cannot be fully avoided.

The distribution of the five *C. sakazakii* serotypes varies geographically and depends also in part on the sources of the isolation. In general, *C. sakazakii* serotypes O1 and O2 are the most frequently occurring *C. sakazakii* serotypes isolated from food and other different environments. For the identification of *C. sakazakii* conventional microbiological cultivation methods consisting of various enrichment procedures and biochemical confirmation are applied (ISO, 2006). These methods are laborious and time-consuming generally requiring 6 days for definitive identification of *C. sakazakii*. Although progress has been made during the last years in the development of highly sensitive and specific, but often complicated and expensive diagnostic methodologies, there is still a lack of a simple and rapid detection method for this pathogen in PIF. Thus, the aim of this study was to establish a multiplexed lateral flow assay, based on specific MAbs, to rapidly and reliably detect the most frequent serotypes of *C. sakazakii*.

In a previous study, we produced a set of specific and sensitive MAbs against *C. sakazakii* and developed a highly specific sandwich-EIA with excellent sensitivity ranging from 2 × 10^3^ to 3 × 10^5^ CFU/ml for the most commonly *C. sakazakii* serotypes O1, O2 as well as the serotype O3. Compared to the ISO standard method, this immunoassay represent a great improvement reducing the time to detect and simultaneous serotype *C. sakazakii* from 6 days to 18 h (Scharinger et al., 2016). Thus, this sandwich-EIA is well-suited for analytical or clinical laboratories but remains a multi-step and laboratory-based procedure. The development of a multiplexed lateral flow detection system to identify *C. sakazakii* contamination would bring additional key advantages, such as (i) lower sample volumes requirement, (ii) portability to enable point-of-care testing, and (iii) easier judgement of results by naked eye. Using the *C. sakazakii* specific MAbs 1C4 (O1) and 2F8 (O2) (Scharinger et al., 2016), we established a novel serotype specific LFA based on the sandwich EIA format. The direct reaction scheme is typically utilized when larger analytes with multiple antigenic sites are tested (Raeisossadati et al., 2016), as it is the case for the surface-located LPS recognized by the *C. sakazakii* serotype specific MAbs. In addition, the developed multiplexed LFA offers the great benefit of high diagnostic efficiency and generates conclusive information relating to *C. sakazakii* serotype. With the new test strip, *C. sakazakii* of the serotypes O1 and O2 can be detected and simultaneously serotyped within 15 min in pure bacterial culture or after an enrichment step of 18 h for environmental and clinical samples or food products.

The practical applicability of the established LFA was thoroughly examined by testing inclusivity and exclusivity, optimal incubation conditions and sensitivity in different baby food matrices. In pure bacterial culture, the lateral flow test strip showed 100% detection for *C. sakazakii* serotypes O1 and O2 and all tested bacteria listed in Table 1. None of the other *Enterobacteriaceae* that often simultaneously occur in the same PIF hampered the analyses and no cross-reactivity with other *Cronobacter* species and *C. sakazakii* serotypes tested was observed, demonstrating the specificity of the developed test system.

For the validation study inoculated PIF samples were prepared at a level which mimics naturally *C. sakazakii* contamination ranges found in infant food formulas. As the level of *Cronobacter* spp. in naturally contaminated samples is with <1 CFU/ml generally very low (Osaili and Forsythe, 2009), the sufficient enrichment of spiked samples up to at least 10^7^ CFU/ml had to be ensured. Prolonged enrichment time increases the final bacterial cell count of the sample and, thus, the detection range of the LFA. According to the time lapses of the ISO standard method, incubation times of up to 20 h in buffered peptone water (BPW) were tested with respect to correlation between found bacterial cell counts and obtained intensity of the LFA test lines. The applicability of the LFA in routine testing was further investigated by the detection of *C. sakazakii* in commercially available PIF reference material and by a comprehensive validation study using three different kinds of infant food formulas for inoculation and three different *C. sakazakii* strains of each serotype at two bacterial cell count levels. In total, 72 different samples (3 baby food types × 2 producer brands × 2 serotypes × 3 strains × 2 CFU levels) were analyzed by LFA. The test strip allowed to detect up to 1–10 CFU in 10 g of infant formula, follow-on formula as well as in baby mash. After subtraction of the samples (*n* = 14), that were negative from the start (i.e., not contaminated), 79.3% (*n* = 46) tested positive for *C. sakazakii* serotypes O1 and O2 already after the initial non-selective enrichment. Negative samples were further enriched in mLST/vancomycin broth for 20 h according to the ISO standard method and were subsequently tested again using the LFA. After this additional enrichment, 96.6% (*n* = 56) of the artificially contaminated samples tested positive. False negative results were obtained only for two samples and in both samples a reduced *Cronobacter* growth was observed, i.e., CFU in non-selective enrichment broth were <6.7 × 10^6^ per ml.

This shows that in the vast majority of cases the LFA successfully detected *C. sakazakii* in PIF samples, inoculated with low (1 CFU/10 g PIF) or high (10 CFU/10 g PIF) pathogen counts, after non-selective or selective enrichment. Thus, the detection for *C. sakazakii* could be shortened from 6 days (ISO standard method) to 1 day (positive test result after non-selective enrichment in BPW) or to 2 days (positive test result after selective enrichment in mLST/vancomycin broth). The detection range of LFA for *C. sakazakii* in artificially contaminated PIF ranged from 10^6^ CFU/ml (in some cases) to 10^7^ CFU/ml for the detection of *C. sakazakii* serotypes O1 and O2, indicating that the LFA retains their sensitivity even in complex matrices such as PIF. Moreover, the detectability was comparable for both serotypes. In comparison to the LOD (≤10^3^ CFU/ml) of the previously described highly specific and sensitive sandwich-EIA (Scharinger et al., 2016), the new multiplexed LFA shows a significantly lower sensitivity, however, the detection range of 10^7^ CFU/ml is comparable to other lateral flow test systems developed for *Cronobacter* (Chen et al., 2014; Song et al., 2016) or other pathogenic bacteria such as *Listeria, Salmonella*, and *Streptococcus* (Raeisossadati et al., 2016; Eltzov and Marks, 2017). It is also noticeable, that after selective enrichment in mLST/vancomycin broth for 10 samples (17.2%, *n* = 10) a *C. sakazakii* contamination could be confirmed which reacted in the first test of the non-selective enrichment negative. This could be simply explained by the fact that, generally, the average of *Cronobacter* cell count in mLST/vancomycin broth was ≥10^8^ CFU/ml, while non-selective enrichment in BPW often showed lower bacterial levels (<6.7 × 10^6^ CFU/ml), which is below the LOD of the developed LFA. Only in plant-based baby food (mash) samples, growth of both *C. sakazakii* serotypes to a higher cell count (10^8^–10^9^ CFU/ml) was consistently observed perhaps because *Cronobacter* is naturally associated with the plant environment as a possible reservoir of this pathogen (Grim et al., 2013; Singh et al., 2015; Vojkovska et al., 2016).

In the validation study, a relatively high percentage (19.4%, *n* = 14) of *Cronobacter* inoculated PIF samples gave negative results both in the LFA and the ISO standard method despite the fact that reconstituted infant formula is considered as a good medium for growth (Parra-Flores et al., 2016). This might be a consequence of spiking PIF samples with very low bacterial cell counts (1 CFU and 10 CFU per 10 g PIF). On the other hand, bacterial cultures are often heterogeneous and characterized by the presence of fast growing and different types of subpopulations–i.e., sensitive, less sensitive, and resistant to different environmental stresses–but not all cells within a population have the same probability to grow. Consequently, the ability of pathogenic microorganisms to grow to a high cell count from a low inoculum size could be affected by individual cell variability (Koutsoumanis, 2008). This variability was recently observed for *C. sakazakii* in single cell and micropopulations of ≤50 cells (Parra-Flores et al., 2016) and could explain false negatives as well as “not contaminated” results in PIF samples spiked with *C. sakazakii* while in pure bacterial cultures (~10^9^ CFU/ml) a 100% detection for *C. sakazakii* serotypes O1 and O2 was achieved.

Advances in developing cost-effective and rapid bacterial test systems using a LFA format, have opened a new field of research in food safety and quality control. Many different LFAs have been developed recently to detect food contaminant compounds such as toxins, bacterial pathogens, or chemical contaminants such as additive, antibiotics (Law et al., 2014; Raeisossadati et al., 2016). During the last years, several immunochromatographic detection strips were developed for *Cronobacter* spp. as well: (i) the detection method of Blažková et al. (2011) combines nucleic acid amplification and the LFA principle but requires two additional steps, namely template DNA isolation and PCR amplification; (ii) Chen et al. (2014) used a silica-coated magnetic nanoparticles separation for developing an immunochromatographic strip; (iii) only Song et al. (2016) developed an immunochromatographic strip assay to specifically identify *C. sakazakii*. The assay was based on rabbit anti-*C. sakazakii* IgG-tagged liposome (immunoliposome) with a LOD of 10^7^ CFU/ml, but the applicability of this test strip for food products was not described. In comparison to these previously described identification methods, the multiplexed LFA realized in this study is significantly easier to perform, less expensive and applicable in food products such as infant food formula. Additionally, the assay allows the simultaneous detection and serotyping of the most frequent *C. sakazakii* serotypes O1 and O2 with a detectability of less than 10^7^ CFU/ml in pure bacterial culture or less than 10 *Cronobacter* cells in 10 g of PIF. In addition, the selected flexible LFA format offers the possibility to expand the number of detection lines so that the detection of other *C. sakazakii* serotypes could be included in future studies. For instance, a MAb enabling the specific detection of *C. sakazakii* serotype O3 has been described recently (Scharinger et al., 2016). Though the LFA was successfully validated in this study and enabled the sensitive detection of *Cronobacter*, particularly in baby food samples, a further increase of the assay sensitivity would be desirable to further improve the detectability of *Cronobacter* in PIF. As the LFA implemented MAbs 1C4 and 2F8 against *C. sakazakii* serotypes O1 and O2 are characterized by a very high affinity (Scharinger et al., 2016), test improvement is not a matter of antibody optimization but rather of signal amplifying strategies (Urusov et al., 2016). For instance, it is possible to considerable increase intensity of test lines and thus reduce the detection range by using gold/silver nanoparticles (Raeisossadati et al., 2016; Oliveira-Rodriguez et al., 2017). An increased sensitivity might improve the applicability of the LFA for on-site analyses.

Overall, the developed LFA can easily be used to routinely test dairy products for *Cronobacter* contamination, and thus ultimately minimize the risk of infant exposure to *Cronobacter* through contaminated PIF. Especially at 35–37°C, *Cronobacter* grows rapidly in PIF. For instance, the pathogen needs 5.2 h at room temperature, 1.8 h at 35°C and ca. 1.3 h at 37°C to grow from 100 to 1,000 CFU (Parra-Flores et al., 2015; K. Schauer, unpublished data). Despite detailed recommendations of the FAO/WHO for the preparation of milk based baby food, e.g., (1) using water at 70°C for PIF reconstitution, (2) the immediate use after being diluted, or (3) if not used, storage at <5°C (FAO/WHO, 2007; Silano et al., 2016), the reconstituted PIF is often stored in a bottle and baby food warmer or at room temperature, which is particularly problematic in geographic areas with high ambient temperature. Here, contaminated PIF with virulent *Cronobacter* could continue to grow and reach high cell densities, which are detectable with the developed LFA. This test strip may be also useful in epidemiological and clinical applications such as monitoring neonatal stations in hospitals for the presence of *C. sakazakii* and analyzing reconstituted PIF or *Cronobacter* isolates for *C. sakazakii* serotypes O1 and O2.

In conclusion, this study presents the first multiplexed LFA suitable for rapid and serotype specific detection of *C. sakazakii* serotypes O1 and O2. This method permits pathogen detection within 15 min in pure culture or after 18 h in PIF after a non-selective enrichment. Given a complete panel of mAbs and further modifications of the system to increase detection sensitivity, all relevant *C. sakazakii* serotypes could be detected with this approach in parallel. Low analysis cost, simplicity and quick performance are making this multiplexed lateral flow test strip a useful tool for point-of-care detection and attractive for fields such as food or health monitoring both to ensure the health of newborns and enhance microbiological safety of PIF.

## Author contributions

KS designed the experiments and wrote the manuscript. ES and KS performed the experiments. R-Biopharm AG and TW developed and prepared lateral flow test strip. KS and RD analyzed and interpreted the data. EM, ES, RD, and TW contributed to the drafting of the manuscript.

### Conflict of interest statement

The authors declare that the research was conducted in the absence of any commercial or financial relationships that could be construed as a potential conflict of interest.
